# Effects of Two Different Irrigation Systems on the Amino Acid Concentrations, Volatile Composition and Sensory Profiles of Godello Musts and Wines

**DOI:** 10.3390/foods8040135

**Published:** 2019-04-22

**Authors:** José Manuel Mirás-Avalos, Yolanda Bouzas-Cid, Emiliano Trigo-Córdoba, Ignacio Orriols, Elena Falqué

**Affiliations:** 1 Estación de Viticultura e Enoloxía de Galicia (EVEGA-AGACAL), Ponte San Clodio s/n, 32428 Leiro–Ourense, Spain; yolanda_maceda@hotmail.com (Y.B.-C.); emilianotrigo@hotmail.com (E.T.-C.); ignacio.orriols.fernandez@xunta.gal (I.O.); 2 Dept. Riego. Centro de Edafología y Biología Aplicada del Segura (CEBAS-CSIC), Campus Universitario de Espinardo, P.O. Box 164, CP 30100 Murcia, Spain; 3 Servizo de Prevención e Análise de Riscos, Dirección Xeral de Innovación e Industrias Agrarias e Forestais, Rúa Roma 25-27, 15703 Santiago de Compostela, Spain; 4 Depto. Química Analítica, Facultad de Ciencias, Universidade de Vigo, As Lagoas s/n, 32004 Ourense, Spain; efalque@uvigo.es

**Keywords:** vine water status, global change, nitrogen compounds, aroma, white wine

## Abstract

The concentrations of amino acids and volatile compounds of a given grapevine cultivar may be modified by climate variability between years and by management practices, such as irrigation, that may alter the typicality of its wines. The current study aimed at assessing the amino acid profile of musts and wines, volatile composition and sensory profile of wines from *Vitis vinifera* (L.) cultivar Godello under rain-fed and two drip irrigation systems (above, drip irrigation (DI), and under the soil surface, subsurface drip irrigation (SDI)) over three consecutive years. Irrigation tended to increase must and wine total acidity; however, it did not alter must amino acid concentrations significantly. Irrigation reduced the concentrations of acetaldehyde and methanol in Godello wines. Moreover, irrigation tended to decrease the concentrations of compounds giving fruity aromas, such as acetaldehyde (by 31% in SDI) and isoamyl acetate (by 21% in SDI), when compared to rain-fed conditions. Sensory analysis revealed slight differences between treatments. Rain-fed and SDI were the treatments showing the greatest differences. Weather conditions affected more must and wine composition than in-season effects caused by irrigation.

## 1. Introduction

Wine aroma is defined by its volatile composition, which depends on many factors including grape variety, climate, soil and vineyard management, amongst others [1,2]. Amino acids in grapes constitute a nitrogen source for yeasts and are responsible for the formation of volatile compounds that define wine aroma, including higher alcohols, volatile fatty acids and ethyl esters [3,4]. These compounds accumulate in grapes during ripening, although their concentrations depend on temperature and water availability [1,5]. Climate change alters the temporal distribution of rainfall and increases the intensity of drought events and heatwaves, raising a great concern in viticulture regions worldwide [6]. In order to counteract these negative effects, irrigation use in vineyards is increasing, even in cool-humid regions [7,8].

Irrigation management can be a tool for modulating berry growth and quality [9]. A great research effort has been devoted to assess the effect of water stress and irrigation scheduling on grapevine (*Vitis vinifera* L.) yield and grape and wine composition, mainly on red cultivars [9,10]. Recently, the effects of water stress on the amino acid and volatile composition of grapes and wines have been determined on white cultivars [11,12,13,14], in which aroma plays an essential role. However, these studies offered contrasting results depending on grapevine cultivar, intensity of water stress, irrigation strategy and climate conditions. 

In the Northwest of the Iberian Peninsula (Galicia and North of Portugal), white grapevine cultivars are predominantly grown and the volatile composition of their grapes and wines might be altered by increasing temperatures and drought events over the growing cycle. Amongst them, Godello provides monovarietal wines recognized worldwide. Volatile composition of Godello wines has already been described [15,16]. Recently, our research group assessed the effect that irrigation might exert on Godello amino acid profile in the Ribeiro Designation of Origin (DO) [17], although only one irrigation strategy was tested and its effects on the wine sensory profile have not been determined.

Therefore, the aim of the current study was to assess the effects of two irrigation systems on the amino acid composition of musts and wines, and the aromatic and sensory profiles of wines from Godello during three years in Northwest Spain. This is a comprehensive approach for studying wine quality since it involves both chemical and sensory evaluations. The obtained results should be of interest to grape growers, winemakers and other wine professionals.

## 2. Materials and Methods 

### 2.1. Studied Vineyard and Experimental Design

The study was carried out from 2012 to 2014 in a Godello vineyard planted in 1997 onto 110-Richter rootstock and located in A Rúa, Ourense, Spain (42° 23’ 59’’ N, 7° 7’ 15’’ W, elevation 320 m). Vines were spaced 1.35 × 1.95 m (3800 vines ha^−1^) and vertically trellised on a double cordon system. Rows were oriented in the north-south direction. Soil is loamy-textured, very acidic and available water capacity is 170 mm m^−1^. According to the multicriteria climatic classification system [18], this area is temperate, moderately dry with cool nights.

The experiment consisted of three treatments on a randomized-block design with three replicates (three rows each, the middle row was used for measurements and sampling, whereas the external rows acted as buffers). The treatments were: Rain-fed (R), surface (DI) and subsurface (SDI) drip irrigation, which reduces direct evaporation from soil and, therefore, more water is available for the vines. The DI pipes were installed on the vineyard row 40 cm above the soil, while SDI pipes were buried 40 cm deep into the soil. Both systems were equipped with 2 L h^−1^ emitters [7], one emitter per vine in the case of DI and one emitter per meter in the case of SDI.

Irrigation treatments started on June 1st and finished in mid-August in 2012; during 2013, irrigation began in July and ended by late August; however, due to the pumping system malfunctioning, irrigation started in mid-July and finished by the end of August. In summary, water was applied for 59, 46 and 34 days in 2012, 2013 and 2014, respectively, at a rate of 1.5 h per day. Average irrigation depths were 1.14 mm and 1.54 mm for DI and SDI, respectively [19]. Agricultural practices were the same for all treatments, except for irrigation.

Several variables were measured over the study period for characterizing the water status, vegetative growth and yield of vines in each treatment. Both methods and results about these determinations are out of the scope of this report and have been described elsewhere [7,19]. However, we summarized the most important findings in the Results and Discussion section.

### 2.2. Sampling and Winemaking

Harvest were performed on the same date, each year, for the three treatments. Samples of 20 kg per field replication were collected for winemaking, one tank was used for two replications; therefore, there were two wines per treatment and year. Two 250 mL must samples from each replicate were collected for determining the general attributes of musts (soluble solids, total acidity, pH) according to official methods after grape pressing [20]. Must samples for determination of amino acids were stored at −4 °C until analysis. 

The winemaking process was the same as that reported previously [13]. In summary, grapes from each replicate were separately destemmed, crushed and pressed (yielding 50% must). Pectolytic enzyme was added (4 g per 100 kg of grape) to favor settling and SO_2_ (50 mg L^−1^) was added to avoid oxidation. After 24 h, musts were racked and, then, fermented in 35 L stainless steel tanks. Commercial yeast (Excellence FW, Lamothe-Abiet, Bordeaux, France) was added (20 g h L^−1^). Once fermentation finished, wines were racked and SO_2_ was added to 35 mg L^−1^ free sulfur dioxide. A natural clarification was performed at 4 °C for one month. Finally, wines were filtered, bottled and stored for five months at 10 °C until analysis. The main attributes of wines were determined according to the official methods [20].

### 2.3. Analytical Determinations

#### 2.3.1. Chemical Reagents

Milli-Q equipment (Millipore, Bedford, MA, USA) allowed for obtaining ultra-pure water. Super-gradient High-Performance Liquid-Chromatography (HPLC) grade acetonitrile and methanol were from Scharlau (Sentmenat, Spain). Ammonium chloride was from Merck (Darmstadt, Germany). Individual L-α amino acids and diethylethoxymethylenemalonate were from Acros Organics (New Jersey, NJ, USA). Amino acid solutions were made with HCl 0.1 N. Dichloromethane, n-pentane and anhydrous sodium sulfate were from Scharlau (Sentmenat, Spain). Standards for volatile compounds were from Merck (Madrid, Spain), Aldrich (Madrid, Spain), Fluka (Seelze, Germany), Alfa Aesar (Barcelona, Spain) and Sigma (Madrid, Spain). All the standards were prepared in 50% hydroalcoholic solutions.

#### 2.3.2. Determination of Free Amino Acids

The determination of amino acids in Godello musts and wines from each treatment was performed by HPLC according to previous works [13,21], using Agilent 1100 series equipment (Agilent Technologies, Palo Alto, CA, USA). Extraction method, reagents, elution conditions and chromatographic separation were performed as described in a previous report [13]. Determinations were carried out in triplicate.

#### 2.3.3. Determination of Volatile Compounds

Concentrations of major volatile compounds were quantified by direct injection in a 7890A gas chromatograph (Agilent Technologies, Palo Alto, CA, USA) [12,16]. Terpenes, C6 alcohols, volatile fatty acids, ethyl esters of fatty acids and acetates of higher alcohols were extracted and injected into a 6890 gas chromatograph (Agilent Technologies, Palo Alto, CA, USA) using the conditions reported in a previous work [13]. Identification was performed using the National Institute of Standards and Technology (NIST) Mass Spectral library by comparing mass spectra and retention times with those of pure standard compounds. All determinations were carried out in triplicate. 

The ratio between concentration of an individual compound and its perception threshold was computed to obtain the odor activity value (OAV) of volatile compounds [22,23].

### 2.4. Sensory Evaluation

A panel of nine judges (five male, four female, age span 30–64) evaluated, each year, the wines from this study. These professionals were oenologists and technicians from Galician wineries, with expertise on assessing white wines, because experienced tasters describe better what they like than consumers or trained panelists [24,25]. For this evaluation, we used a scorecard consisting of 21 descriptors (four for color, 10 for aroma, and seven for palate), which were specifically chosen for Galician white wines and were scored from 0 (not present) to 9 (very intense) [26,27]. Apart from the fruity descriptors such as dry fruit or tropical fruit, quantitative descriptors such as persistence (duration of the perception of aroma) and intensity were included in this scorecard. Furthermore, tasters scored the global quality of the wine. Wine samples (30 mL) coded with three random numbers were presented in clear tulip-shaped glasses. The tasting sessions took place in different days (April and May from the year after the corresponding vintage). 

### 2.5. Statistical Analysis

The effects of year, irrigation treatment and their interaction on amino acids and volatile compound concentrations were assessed by analysis of variance (ANOVA). When required, mean separation was performed using the Tukey’s test. A principal component analysis (PCA) was carried out to separate must samples according to their amino acid concentrations and wines according to their volatile and sensory profiles. Statistical analyses were performed using R v3.2.2 [28]. Data from the sensory evaluation were processed using Big Sensory Soft 1.02 (Centro Studi Assagiatori, Brescia, Italy), using the non-parametric Friedman test to discern which descriptors differed between treatments since these data do not comply with the assumption of normality [29].

## 3. Results and Discussion

### 3.1. Weather Conditions and Vine Water Status

A summary of the weather conditions over the study period (2012–2014) is presented in Table 1. Annual rainfall was lower in 2012 than in the other studied years. However, rainfall over the growing season (April to harvest) was 260 mm, 331 mm and 239 mm in 2012, 2013 and 2014, respectively (Table 1). During maturation (veraison to harvest), temperatures were similar among the studied years; however, the rainfall pattern differed. Annual mean temperature was higher in 2014 than in 2012 and 2013. Mean temperatures over the growing season increased from year to year, and reference evapotranspiration (ET_o_) was greater in 2013 than in the other studied years (Table 1).

Previous studies reported data on the effects of irrigation on physiology and yield response of vines in this site [7,19]. In summary, rain-fed vines showed more negative midday stem water potentials than those irrigated; whereas yield and cluster weight were similar among treatments (Table 2). Mild water stress was observed in both rain-fed and irrigated vines in 2012; while rain-fed vines suffered from mild water stress also in 2013 (Table 2). No stress conditions were observed in 2014. In summary, rain-fed vines showed more negative stem water potentials than those irrigated, especially from veraison onwards; these differences were more marked in 2013, the warmest growing season [19]. This explains the absence of significant differences among treatments in yield. In fact, only the number of clusters per vine differed significantly among treatments in 2013, when the vines from SDI had a greater number of clusters than those from the other treatments (Table 2).

### 3.2. General Parameters of Musts and Wines

Year exerted a significant effect on tartaric acid, whereas treatment did not affect any of the considered attributes, although a trend (*p* < 0.1) to higher acidities in musts from the irrigated treatments was observed (Table 3). No significant interactions between factors were detected. Acid-balanced wines usually have refreshing or crisp sensory undertones because organic acids, such as tartaric, play important roles in the development of specific flavor compounds [30]. 

Vines in our study experienced a weak level of water stress (Table 2) and this prevents to observe changes in must attributes [31]. However, total acidity showed a great sensitivity to irrigation, as previously observed for other cultivars [8,9,14]. These slight changes might be not sufficient to detect differences among treatments in the analytical determinations carried out; however, a year-to-year variability existed in the general attributes of musts. For instance, up to 2 °Brix difference between grapes harvested in 2013 and 2014, accompanied by a 1 g L^−1^ difference in total acidity and about 1.5 g L^−1^ in tartaric acid concentration (Table 3). This different maturation can be explained by rainfall and temperature patterns over the ripening period in each year, exerting a great effect on musts and wine quality [1]. 

In wines, both year and treatment significantly altered total acidity and tartaric acid concentrations, following the trends detected in musts. Wines from SDI showed the highest TA and the lowest pH values (Table 4). This response is common in white cultivars under weak or mild stress conditions [7,8,32], and might have been caused by higher water availability over the maturation period in the irrigated treatments, especially in SDI.

### 3.3. Amino Acids Profiles of Musts and Wines

The amino acid concentrations in Godello musts (Table 5) were within previously reported levels, except for proline that appeared at concentrations lower than those previously reported for other cultivars [3]. Proline is accumulated in grapes when vines suffer from water stress [12,33], which was not the case in our study (Table 2). The concentrations of amino acids observed in the current study were very similar to those reported for Godello in a previous work despite the different rootstock, soil and weather conditions between the experiments in both studies [17]. This agrees with the hypothesis that amino acid profile is relatively constant for a given variety [21]. 

In this sense, Godello is an arginine accumulator as this amino acid accounted for 25% of the total free amino acids in musts, similarly to other cultivars, such as Syrah, Petit Verdot and Merlot [21]. Other amino acids present at relevant concentrations in Godello musts were glutamic acid (14%), alanine (10%) and glutamine (9%); abundant in other Spanish white varieties, such as Verdejo and Albariño [11,34]. In our study, irrigation did not modify the amino acid profile of this variety over the three years studied. 

In the current study, irrigation did not affect the amino acid concentrations of Godello musts except for reducing that of tryptophan in 2013 (Table 5). Although not significantly, SDI tended to reduce glutamic acid (*p* = 0.07) and valine (*p* = 0.06) concentrations when compared to the rain-fed control in 2012. In contrast, the effect of year was significant for aspartic and glutamic acids, asparagine, serine, histidine, threonine, γ-aminobutyric acid (GABA), tyrosine, cysteine, tryptophan, ornithine and lysine, and the sum of amino acid concentrations in Godello musts (Table 5). No significant interactions between treatment and year were detected. As previously stated, rainfall and temperature regimes between veraison and harvest differed among the studied years, being 2013 a drier year than 2012 and 2014, likely causing significant variations in the concentrations of amino acids in Godello musts, as these compounds are strongly affected by weather conditions during berry maturation [1]. In contrast, the weak water deficit suffered by rain-fed vines between veraison and harvest compared to irrigated vines (Table 2) was not enough to alter must amino acid profiles.

**Figure 1:**
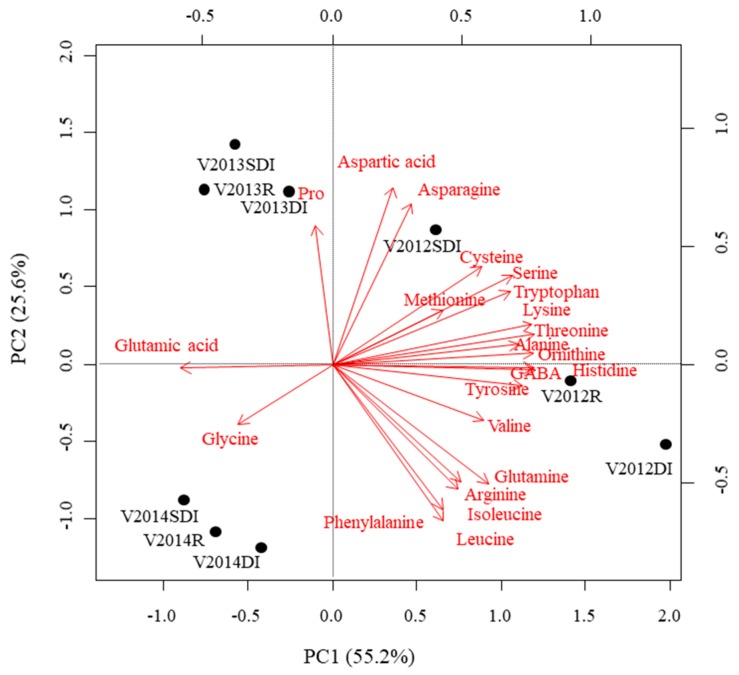
Principal component analysis (PCA) of Godello musts: Biplot for the first two principal components (PC) for the concentrations of free amino acids in musts. R = Rain-fed, DI = Drip irrigation, SDI = Subsurface drip irrigation.

This was confirmed by a PCA ran on the covariance matrix of the concentrations of the free amino acids in must. The two first principal components (PC) explained 80.8% of the total variance in the dataset (Figure 1). Samples separated according to year, whereas irrigation treatment had a lower influence. Musts from 2012 were on the positive side of PC1, due to high concentrations of most amino acids, including asparagine, GABA, histidine, valine, alanine, etc. Musts from 2013 were on the positive side of PC2 but on the negative side of PC1 due to their high concentrations of proline. Samples from 2014 were on the negative side of both PC due to their high concentrations of glycine. In the case of 2012 samples, PCA showed a separation according to treatment, samples from R treatment had higher concentrations of GABA, histidine, tyrosine, alanine and ornithine, whereas samples from DI had high concentrations of valine and those from SDI had higher concentrations of asparagine and cysteine (Figure 1).

In agreement with a previous report on the cultivar Verdejo [11], our results showed that irrigation did not affect amino acid concentrations in Godello musts, except for some individual compounds in a given year, being weather conditions and maturation stage the main drivers for determining the concentrations of amino acids in the musts. In our study, the highest amino acid concentrations in Godello musts were observed in 2012 [7], the year with the most negative midday stem water potentials (Table 2). Weather conditions can cause a great variation in the concentrations of individual amino acids from year to year, although their relative proportions over the total free amino acids remain constant [11,34].

**Figure 2:**
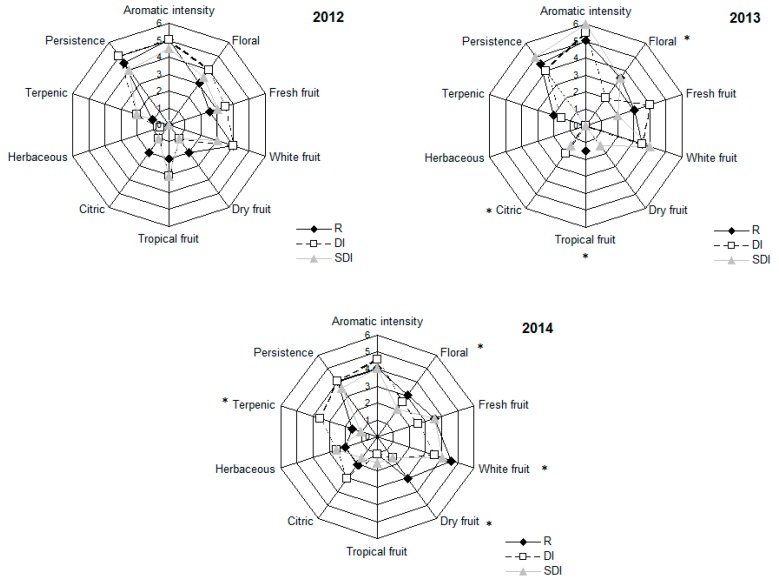
Aroma descriptors for Godello wines (from 2012 to 2014) as a function of rain-fed and irrigation conditions. R = Rain-fed, DI = Drip irrigation, SDI = Subsurface drip irrigation. Asterisks indicate significant differences among treatments according to the Friedman’s test.

Although previous research showed that the percentage of amino acids allowed for a differentiation of grapes according to the variety and cultivation system [21], regardless of the year, other studies revealed a great inter-annual variation on the amino acid composition of grapes and musts [11]. This is directly related to the weather conditions of each year. In the current study, mean temperature increased from year to year; however, evapotranspiration was higher in 2013. In contrast, rainfall distribution was different among years. In general, 2012 could be considered a cooler and drier year (Table 1). Likely, this has caused that samples were more different between years than between irrigation treatments (Figure 2).

In the case of wines, those from SDI showed the lowest concentrations of serine, glutamine, glycine and isoleucine, as well as the lowest sum of free amino acids (Supplementary Table S1). Year exerted a significant influence on the concentration of 15 individual compounds and on the total concentration of free amino acids in Godello wines. No significant interactions between treatment and year were detected. These results were similar to those from a previous work [17].

### 3.4. Volatile Composition of Godello Wines

Volatile compounds in the wines from the current study (Table 6) were present at similar concentrations to those previously reported for Godello wines [15,16]. Irrigation tended to reduce the concentrations of methanol, acetaldehyde, acetoine, acetol and citronellol (Table 6). In addition, year affected most of the volatile compounds determined in Godello wines (Table 6). Significant interactions between treatment and year were observed for benzyl alcohol, acetol and hodiol I.

The effect of the treatments differed among years. In 2012, higher concentrations of benzyl alcohol, acetoine and acetol appeared in wines from R, whereas 2-phenylethyl acetate was at lower concentrations in wines from SDI. In 2013, 1-propanol, 3-methyl-1-butanol and the sum of higher alcohols were greater in wines from DI, whereas the concentrations of *trans*-linalool-oxide (pyran) and citronellol were higher in wines from R. In 2014, wines from SDI had higher concentrations of benzyl alcohol, ethyl lactate, isobutyric acid and the sum of short-chained fatty acids, whereas wines from R had higher concentrations of citronellol and hodiol I (Table 6). Previous reports on the effect of irrigation on wine volatile composition offered contrasting results depending on grapevine variety, soil type, climate conditions and severity and time of occurrence of water stress [10,14,17]. For instance, Talaverano et al. [10] reported higher concentrations of alcohols, C6 compounds and volatile phenols in Tempranillo wines from rain-fed vines. Similarly, Vilanova et al. [14] observed a reduction in C6 compounds in Verdejo wines from irrigated vines. These findings contrasted with the results from the current study likely because of the different cultivar/rootstock combination and climate conditions. In fact, soil water availability was adequate for bud-break and the development of leaf surface in our study [7,19], so no differences in grapevine metabolism were expected.

In this study, 19 volatile compounds appeared in wines at concentrations higher than their odor thresholds (Table 7), likely contributing to wine aroma [22,23]. Acetaldehyde, 1-propanol, isoamyl acetate, ethyl hexanoate, ethyl octanoate and isovaleric acid were present at concentrations more than 15 times higher than their respective odor thresholds, being consistent with previous studies [16,17]. Irrigation did not affect OAV of any compound, except on specific years (Table 7). For instance, OAV for 1-propanol was higher for the DI treatment in 2013, whereas that of benzyl alcohol was higher for SDI in 2014 (Table 7).

### 3.5. Sensory Profiles of Godello Wines

No clear differences among treatments were detected for color (Supplementary Figure S1). Aroma descriptors (Figure 2) were similar for the three treatments in 2012 and 2013; however, wines from R received greater marks to fresh, dry and tropical fruit descriptors than wines from the irrigated treatments, especially those from SDI (Figure 2). Palate descriptors did not reveal a clear preference of the tasters for wines from a given treatment (Supplementary Figure S1). As reported for other white varieties [8], Godello wines from irrigated vines were more acidic but showed an aroma complexity similar to that of wines from the rain-fed treatment. Overall, the slight differences observed in the sensory characteristics were likely caused by the low level of water stress experienced by grapevines [7], which did not affect the Godello vine performance.

**Figure 3:**
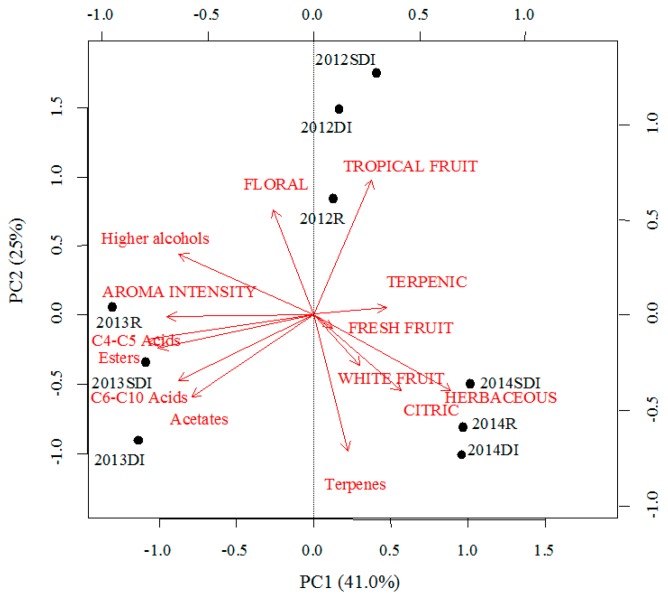
Principal component analysis (PCA) of Godello wines: Biplot for the first two principal components (PC) for aroma descriptors (in upper-case letters) and the different families of volatile compounds quantified (in lower-case letters). R = Rain-fed, DI = Drip irrigation, SDI = Subsurface drip irrigation.

When applying a PCA to the aroma descriptors and the families of volatile compounds quantified in Godello wines (Figure 3), the first two PC explained 66% of the variability in the samples. PC1 explained 41% of the variance and was positively related to the citric and herbaceous descriptors, and negatively associated to aroma intensity and the concentration of volatile fatty acids, higher alcohols and esters. The PC2 explained 25% of the variance and was positively related to tropical fruit and floral descriptors, and negatively related to the concentration of terpenes. The projection of the samples on this plane allowed for a clear differentiation of the studied years, but not by treatments. Wines from 2012 were on the positive side of both PC due to their marks for tropical fruit and floral descriptors. In contrast, wines from 2013 had greater concentrations of higher alcohols and volatile fatty acids, appearing on the negative side of both PC. Finally, wines from 2014 were on the positive side of PC1 due to their greater concentrations of free terpenes and higher marks for citric and herbaceous descriptors. Aggrupation of wines from the same year is consistent with other reports [35], proving that in-season effects caused by irrigation were less important than the influence of weather conditions.

## 4. Conclusions

Under the climate conditions of Northwest Iberian Peninsula, irrigation had a low incidence on amino acid and volatile composition of Godello musts and wines, because of the weak level of water stress experienced by vines from veraison to harvest. Despite this, rain-fed vines produced wines with lower acidity and higher pH than those from the treatment under subsurface drip irrigation. Moreover, irrigation decreased the concentration of volatile compounds providing fruity aromas, such as acetaldehyde and isoamyl acetate, leading to slight differences in wine perception by tasters. 

## Figures and Tables

**Table 1:** Mean temperature, potential evapotranspiration (ET_o_) and rainfall over the growing season (April to harvest), and annual rainfall and mean temperature of each year (2012–2014) in the studied vineyard.

Year	Growing Season Rainfall (mm)	Annual Rainfall (mm)	Growing Season Mean Temperature (°C)	Annual Mean Temperature (°C)	Growing Season ET_o_ (mm)
2012	260	543	16.8	12.7	706
2013	331	926	17.1	12.7	741
2014	239	825	17.4	13.4	698

**Table 2:** Water status, number of clusters, yield and cluster weight of Godello vines subjected to rain-fed and irrigation conditions during three consecutive seasons (2012, 2013, and 2014).

Year	Treatment	Midday Stem Water Potential from Veraison to Harvest (MPa)	Clusters per Vine	Yield (kg vine^−1^)	Cluster Weight (g cluster^−1^)
2012	R	−0.93 b	22.2	2.8	130.3
	DI	−0.85 ab	19.4	2.9	145.0
	SDI	−0.80 a	21.6	3.5	152.5
2013	R	−0.91 b	21.3 a	3.2	142.7
	DI	−0.78 a	20.8 a	3.7	171.0
	SDI	−0.71 a	26.7 b	4.2	152.8
2014	R	−0.72 b	22.9	2.9	122.1
	DI	−0.62 a	21.4	2.7	125.0
	SDI	−0.58 a	19.3	2.4	117.7

For each year, different letters indicate significant differences between treatments at *p* < 0.05. R = rain-fed; DI = drip irrigation; SDI = subsurface drip irrigation.

**Table 3:** General attributes of the musts (mean ± standard error) of Godello vines under three irrigation regimes over three seasons (2012, 2013, and 2014). The significances of the factors year and treatment, as well as their interaction are shown.

Parameter	2012			2013			2014			T	Y	T × Y
	R	DI	SDI	R	DI	SDI	R	DI	SDI			
**Total soluble solids (°Brix)**	23.3 ± 0.1	22.8 ± 0.1	22.2 ± 0.4	24.3 ± 0.2	24.1 ± 0.6	24.0 ± 0.3	22.0 ± 0.0	21.7 ± 0.1	21.7 ± 0.2	ns	ns	ns
**Total acidity (g L^−1^)**	6.5 ± 0.7	7.0 ± 0.2	7.9 ± 0.6	6.2 ± 0.2	6.1 ± 0.1	6.9 ± 0.5	7.1 ± 0.6	7.4 ± 0.3	7.9 ± 0.2	ns	ns	ns
**pH**	3.20 ± 0.02	3.18 ± 0.01	3.14 ± 0.01	3.33 ± 0.01	3.33 ± 0.02	3.26 ± 0.06	3.17 ± 0.05	3.13 ± 0.04	3.06 ± 0.02	ns	ns	ns
**Malic acid (g L^−1^)**	4.0 ± 1.4	3.4 ± 0.2	4.1 ± 0.3	2.8 ± 0.3	2.9 ± 0.1	3.1 ± 0.1	3.0 ± 0.3	3.1 ± 0.2	3.2 ± 0.1	ns	ns	ns
**Tartaric acid (g L^−1^)**	5.7 ± 0.1	4.7 ± 0.8	6.3 ± 0.4	6.6 ± 0.0	6.3 ± 0.2	6.8 ± 0.4	8.2 ± 0.4	8.2 ± 0.3	8.6 ± 0.4	ns	***	ns

For each year, different letters indicate significant differences between treatments at *p* < 0.05. The significance of the year, treatment and their interaction is expressed as ns = not significant; *** *p* < 0.001. R = rain-fed; DI = drip irrigation; SDI = subsurface drip irrigation; T = Treatment; Y = Year.

**Table 4:** General attributes of the wines (mean ± standard error) from Godello vines under three irrigation regimes over three seasons (2012, 2013, and 2014). The significances of the factors year and treatment, as well as their interaction are shown.

Parameter	2012			2013			2014			T	Y	T × Y
	R	DI	SDI	R	DI	SDI	R	DI	SDI			
**Alcohol (%vol.)**	14.1 ± 0.1	14.0 ± 0.2	13.6 ± 0.5	14.0 ± 0.2	14.2 ± 0.1	14.1 ± 0.0	14.3 ± 0.2	14.3 ± 0.1	14.0 ± 0.0	ns	ns	ns
**Total acidity (g L^−1^)**	**7.0 ± 0.1 ab**	**6.7 ± 0.1 a**	**7.6 ± 0.2 b**	**7.1 ± 0.2 a**	**7.5 ± 0.2 a**	**8.0 ± 0.3 b**	7.9 ± 0.3	8.1 ± 0.2	8.7 ± 0.3	***	***	ns
**pH**	3.26 ± 0.02	3.26 ± 0.02	3.12 ± 0.06	3.37 ± 0.08	3.39 ± 0.01	3.26 ± 0.06	**3.18 ± 0.01 b**	**3.09 ± 0.09 ab**	**2.89 ± 0.01 a**	ns	ns	ns
**Malic acid (g L^−1^)**	2.4 ± 0.3	2.4 ± 0.2	2.7 ± 0.1	2.5 ± 0.3	2.9 ± 0.1	2.7 ± 0.1	2.3 ± 0.2	2.4 ± 0.1	2.2 ± 0.2	ns	ns	ns
**Tartaric acid (g L^−1^)**	2.2 ± 0.3	2.4 ± 0.1	2.8 ± 0.4	2.9 ± 0.3	2.7 ± 0.1	3.3 ± 0.3	**4.2 ± 0.2 a**	**4.3 ± 0.4 a**	**5.6 ± 0.3 b**	*	***	ns

For each year, different letters indicate significant differences between treatments at *p* < 0.05. The significance of the year, treatment and their interaction is expressed as ns = not significant; * *p* < 0.05; *** *p* < 0.001. Bold letters indicate significant differences among treatments for a given parameter and year. R = rain-fed; DI = drip irrigation; SDI = subsurface drip irrigation; T = Treatment; Y = Year.

**Table 5:** Irrigation effects on the amino acid concentrations (mean ± standard error, mg L^−1^) of Godello musts. The significances of the factors year and treatment, as well as their interaction are shown. R = rain-fed, DI = drip irrigation, SDI = subsurface drip irrigation, T = Treatment, Y = Year.

Compound	2012			2013			2014			T	Y	T × Y
	R	DI	SDI	R	DI	SDI	R	DI	SDI			
**Aspartic acid**	42.5 ± 8.5	44.5 ± 5.9	54.1 ± 0.1	51.7 ± 0.8	59.8 ± 3.1	58.7 ± 1.9	22.2 ± 3.8	24.7 ± 1.7	25.3 ± 2.1	ns	*	ns
**Glutamic acid**	75.7 ± 10.0	60.3 ± 4.8	42.7 ± 1.9	95.7 ± 2.3	101.9 ± 4.4	100.4 ± 3.0	102.8 ± 10.7	96.5 ± 2.9	89.6 ± 2.3	ns	**	ns
**Asparagine**	3.2 ± 0.7	3.0 ± 1.3	2.8 ± 1.0	3.1 ± 0.6	3.4 ± 0.2	4.2 ± 0.5	1.3 ± 0.3	1.3 ± 0.0	1.7 ± 0.4	ns	*	ns
**Serine**	44.4 ± 3.8	47.9 ± 7.5	40.8 ± 7.3	36.7 ± 3.7	39.5 ± 4.6	38.3 ± 0.5	26.6 ± 5.3	28.4 ± 0.9	26.7 ± 1.2	ns	***	ns
**Glutamine**	61.3 ± 8.9	69.5 ± 24.5	55.1 ± 11.0	43.6 ± 6.6	43.7 ± 7.0	41.3 ± 2.3	54.9 ± 17.8	56.0 ± 1.5	49.6 ± 4.1	ns	ns	ns
**Histidine**	18.7 ± 3.3	17.5 ± 4.1	12.9 ± 2.7	10.0 ± 1.3	10.9 ± 1.6	10.0 ± 0.4	8.9 ± 1.9	9.5 ± 0.1	8.8 ± 1.0	ns	**	ns
**Glycine**	2.5 ± 0.3	2.6 ± 0.3	2.0 ± 0.6	2.7 ± 0.3	2.8 ± 0.5	2.7 ± 0.0	2.6 ± 0.3	3.0 ± 0.0	2.8 ± 0.0	ns	ns	ns
**Threonine**	79.8 ± 13.1	85.6 ± 19.7	74.4 ± 13.7	55.3 ± 7.2	54.6 ± 7.5	54.4 ± 2.3	44.0 ± 11.5	46.7 ± 1.4	44.9 ± 3.3	ns	***	ns
**Arginine**	153.9 ± 23.8	245.6 ± 88.3	148.7 ± 41.9	120.4 ± 21.3	127.9 ± 16.8	126.5 ± 2.4	157.7 ± 47.1	170.0 ± 2.9	159.6 ± 20.6	ns	ns	ns
**Alanine**	62.4 ± 15.9	78.6 ± 27.8	57.1 ± 16.5	51.0 ± 11.1	57.8 ± 12.8	56.5 ± 4.1	46.5 ± 13.4	51.9 ± 3.7	43.5 ± 3.4	ns	ns	ns
**γ-Aminobutyric acid (GABA)**	102.1 ± 4.3	111.3 ± 4.7	82.8 ± 18.9	30.0 ± 3.7	32.8 ± 6.9	28.6 ± 1.7	26.6 ± 3.0	26.5 ± 1.2	20.6 ± 1.8	ns	***	ns
**Proline**	4.9 ± 1.1	3.5 ± 1.9	2.2 ± 1.3	6.4 ± 0.0	6.1 ± 0.4	6.7 ± 1.0	3.2 ± 0.4	2.7 ± 0.2	2.7 ± 0.2	ns	ns	ns
**Tyrosine**	3.0 ± 0.3	3.2 ± 0.5	2.8 ± 0.1	2.5 ± 0.6	2.3 ± 0.3	1.9 ± 0.5	2.0 ± 0.2	2.4 ± 0.1	2.2 ± 0.1	ns	*	ns
**Ammonium ion**	124.3 ± 19.4	124.1 ± 21.9	120.9 ± 1.1	142.4 ± 3.4	148.6 ± 10.9	149.1 ± 13.2	117.0 ± 23.0	132.1 ± 6.4	127.3 ± 9.4	ns	ns	ns
**Valine**	18.8 ± 1.5	17.9 ± 1.4	12.0 ± 2.2	13.6 ± 0.7	14.7 ± 2.7	13.4 ± 0.4	13.9 ± 1.4	14.8 ± 0.1	13.2 ± 0.6	ns	ns	ns
**Methionine**	1.6 ± 0.2	1.9 ± 0.6	1.2 ± 0.2	1.7 ± 0.2	1.5 ± 0.0	1.7 ± 0.0	1.2 ± 0.2	1.5 ± 0.2	1.3 ± 0.1	ns	ns	ns
**Cysteine**	2.5 ± 0.2	2.3 ± 0.2	2.6 ± 0.0	2.2 ± 0.1	2.3 ± 0.0	2.1 ± 0.1	1.9 ± 0.0	1.9 ± 0.1	1.9 ± 0.1	ns	***	ns
**Isoleucine**	8.8 ± 1.0	7.9 ± 0.9	6.0 ± 1.0	5.7 ± 0.3	6.1 ± 1.1	5.8 ± 0.1	7.0 ± 0.8	7.4 ± 0.1	6.7 ± 0.4	ns	ns	ns
**Tryptophan**	5.7 ± 0.2	5.3 ± 2.3	5.5 ± 0.3	**4.3 ± 0.2 b**	**3.7 ± 0.0 ab**	**2.7 ± 0.3 a**	2.1 ± 0.3	2.2 ± 0.2	1.5 ± 0.1	ns	***	ns
**Leucine**	10.3 ± 0.9	11.1 ± 1.5	6.9 ± 1.4	6.0 ± 0.6	7.4 ± 1.4	7.3 ± 0.4	9.2 ± 0.7	9.2 ± 0.0	8.8 ± 0.8	ns	ns	ns
**Phenylalanine**	8.0 ± 0.3	8.3 ± 1.8	6.1 ± 1.2	5.5 ± 0.7	5.8 ± 0.9	5.5 ± 0.1	7.1 ± 0.9	7.6 ± 0.0	7.1 ± 0.1	ns	ns	ns
**Ornithine**	1.1 ± 0.3	1.6 ± 0.7	1.0 ± 0.2	0.7 ± 0.2	0.7 ± 0.1	0.7 ± 0.0	0.5 ± 0.1	0.5 ± 0.0	0.5 ± 0.0	ns	**	ns
**Lysine**	3.7 ± 0.6	3.9 ± 1.0	3.1 ± 0.7	2.6 ± 0.5	2.8 ± 0.4	2.8 ± 0.1	2.1 ± 0.3	2.2 ± 0.1	2.1 ± 0.2	ns	**	ns
**Sum of amino acids**	714.9 ± 92.4	833.3 ± 201.1	622.6 ± 123.8	551.1 ± 62.7	588.4 ± 66.7	572.0 ± 11.6	544.2 ± 114.2	566.5 ± 6.9	520.7 ± 39.0	ns	*	ns

Different letters indicate significant differences between treatments at *p* < 0.05 for a given amino acid in a given year; for a rapid identification, these values appear in bold. The significance of the year, treatment and their interaction is expressed as ns = non-significant; * *p* < 0.05; ** *p* < 0.01; *** *p* < 0.001.

**Table 6:** Irrigation effects on the concentrations of volatile compounds (mean ± standard error) of Godello wines. The significances of the year, treatment as well as their interaction are also shown. R = rain-fed, DI = drip irrigation, SDI = subsurface drip irrigation, T = Treatment, Y = Year.

	2012			2013			2014			T	Y	T × Y
	R	DI	SDI	R	DI	SDI	R	DI	SDI			
Methanol (mg L−1)	21 ± 1	18 ± 1	17 ± 0	19 ± 1	20 ± 1	18 ± 0	22 ± 3	19 ± 1	18 ± 1	*	ns	ns
Ethyl acetate (mg L−1)	59 ± 11	56 ± 5	59 ± 1	26 ± 2	26 ± 1	27 ± 3	37 ± 6	34 ± 1	38 ± 0	ns	*	ns
Acetaldehyde (mg L−1)	58 ± 12	39 ± 1	28 ± 2	31 ± 3	35 ± 3	30 ± 2	31 ± 6	28 ± 0	25 ± 0	*	**	ns
**Higher alcohols (mg L^−1^)**												
1-propanol	12 ± 0	13 ± 1	12 ± 2	**22 ± 1 ab**	**24 ± 0 b**	**20 ± 0 a**	23 ± 4	24 ± 4	17 ± 2	ns	**	ns
2-methyl-1-propanol	61 ± 5	60 ± 3	69 ± 7	35 ± 2	37 ± 1	34 ± 0	28 ± 6	24 ± 3	45 ± 8	ns	***	ns
2-methyl-1-butanol	67 ± 4	64 ± 1	67 ± 1	64 ± 1	72 ± 1	64 ± 3	42 ± 4	44 ± 4	50 ± 1	ns	***	ns
3-methyl-1-butanol	252 ± 23	261 ± 19	275 ± 4	**289 ± 6 a**	**313 ± 2 b**	**286 ± 0 a**	221 ± 8	219 ± 8	253 ± 15	ns	ns	ns
∑ Higher alcohols	392 ± 21	398 ± 23	423 ± 12	**409 ± 8 a**	**446 ± 2 b**	**403 ± 3 a**	314 ± 14	311 ± 11	365 ± 21	ns	**	ns
**Other alcohols (mg L^−1^)**												
1-hexanol	1.8 ± 0.1	1.7 ± 0.8	2.3 ± 0.4	1.8 ± 0.0	1.8 ± 0.2	1.8 ± 0.0	2.0 ± 0.2	2.0 ± 0.1	2.1 ± 0.1	ns	ns	ns
trans-3-hexen-1-ol	0.26 ± 0.06	0.34 ± 0.08	0.16 ± 0.02	0.14 ± 0.00	0.13 ± 0.00	0.12 ± 0.00	0.15 ± 0.01	0.15 ± 0.00	0.12 ± 0.03	ns	*	ns
cis-3-hexen-1-ol	0.54 ± 0.10	0.52 ± 0.01	0.27 ± 0.03	0.21 ± 0.02	0.22 ± 0.01	0.23 ± 0.03	0.22 ± 0.00	0.23 ± 0.01	0.18 ± 0.05	ns	**	ns
Benzyl alcohol	**3.82 ± 0.06 b**	**2.73 ± 0.03 a**	**2.77 ± 0.04 a**	2.28 ± 0.14	2.47 ± 0.13	2.15 ± 0.11	**2.12 ± 0.08 a**	**2.48 ± 0.03 ab**	**2.79 ± 0.11 b**	ns	**	**
2-phenylethanol	56 ± 2	50 ± 4	43 ± 5	48 ± 4	57 ± 1	50 ± 4	**34 ± 1 ab**	**38 ± 0 b**	**33 ± 1 a**	ns	**	ns
**Other compounds (mg L^−1^)**												
Ethyl lactate	14 ± 1	10 ± 0	12 ± 2	5 ± 0	6 ± 0	6 ± 0	**4 ± 0 a**	**5 ± 0 ab**	**6 ± 0 b**	ns	***	ns
Acetoine	**8 ± 1 b**	**4 ± 1 a**	**3 ± 0 a**	3 ± 0	3 ± 0	3 ± 0	4 ± 2	3 ± 2	<LOD	*	*	ns
Acetol	**92 ± 0 b**	**34 ± 6 a**	**30 ± 2 a**	33 ± 3	35 ± 1	28 ± 1	10 ± 4	8 ± 0	11 ± 1	**	***	***
2,3-butanediol levo	729 ± 116	776 ± 51	618 ± 54	712 ± 11	691 ± 29	692 ± 13	1565 ± 367	1552 ± 422	733 ± 110	ns	**	ns
2,3-butanediol meso	332 ± 17	332 ± 15	317 ± 13	352 ± 3	350 ± 6	347 ± 8	318 ± 81	314 ± 90	130 ± 28	ns	ns	ns
Methionol	0.78 ± 0.05	0.82 ± 0.14	0.67 ± 0.10	0.41 ± 0.04	0.49 ± 0.06	0.44 ± 0.06	0.25 ± 0.05	0.22 ± 0.04	0.43 ± 0.07	ns	***	ns
**Acetates of higher alcohols (mg L^−1^)**												
Isoamyl acetate	0.71 ± 0.03	0.67 ± 0.04	0.51 ± 0.10	1.25 ± 0.38	1.34 ± 0.35	0.76 ± 0.01	0.74 ± 0.09	0.83 ± 0.03	0.86 ± 0.06	ns	ns	ns
Hexyl acetate	0.54 ± 0.21	0.36 ± 0.08	0.22 ± 0.16	0.26 ± 0.02	0.37 ± 0.04	0.42 ± 0.05	0.29 ± 0.04	0.22 ± 0.02	0.25 ± 0.01	ns	ns	ns
2-phenylethyl acetate	**0.04 ± 0.00 ab**	**0.05 ± 0.00 b**	**0.03 ± 0.00 a**	0.25 ± 0.01	0.22 ± 0.03	0.20 ± 0.01	0.08 ± 0.03	0.09 ± 0.01	0.06 ± 0.01	ns	ns	ns
∑ Acetates	1.29 ± 0.20	1.08 ± 0.18	0.76 ± 0.14	1.76 ± 0.33	1.93 ± 0.35	1.38 ± 0.16	1.11 ± 0.20	1.14 ± 0.23	1.17 ± 0.24	ns	ns	ns
**Esters (mg L^−1^)**												
Ethyl butyrate	0.04 ± 0.00	0.04 ± 0.00	0.03 ± 0.02	< LOD	< LOD	< LOD	0.05 ± 0.00	0.05 ± 0.00	0.06 ± 0.00	ns	*	ns
Ethyl hexanoate	0.26 ± 0.01	0.29 ± 0.01	0.30 ± 0.09	0.44 ± 0.03	0.36 ± 0.01	0.39 ± 0.02	0.24 ± 0.02	0.25 ± 0.00	0.22 ± 0.00	ns	ns	ns
Ethyl octanoate	0.34 ± 0.00	0.41 ± 0.01	0.38 ± 0.08	0.71 ± 0.07	0.54 ± 0.07	0.60 ± 0.11	0.29 ± 0.00	0.34 ± 0.01	0.32 ± 0.04	ns	ns	ns
Ethyl decanoate	0.08 ± 0.02	0.09 ± 0.01	0.08 ± 0.01	0.30 ± 0.03	0.28 ± 0.05	0.29 ± 0.03	0.11 ± 0.01	0.12 ± 0.00	0.12 ± 0.02	ns	ns	ns
∑ Ethyl esters C6-C10	0.72 ± 0.03	0.83 ± 0.03	0.79 ± 0.21	1.45 ± 0.13	1.18 ± 0.13	1.29 ± 0.16	0.70 ± 0.02	0.76 ± 0.01	0.72 ± 0.06	ns	ns	ns
**Volatile fatty acids (mg L^−1^)**												
Isobutyric acid	3.40 ± 0.47	2.78 ± 0.17	2.76 ± 0.11	3.87 ± 0.30	4.15 ± 0.40	3.65 ± 0.15	**1.76 ± 0.17 a**	**1.53 ± 0.10 a**	2.48 ± 0.14 b	ns	*	ns
Butyric acid	0.98 ± 0.11	0.92 ± 0.00	0.87 ± 0.08	1.48 ± 0.03	1.46 ± 0.03	1.37 ± 0.04	0.85 ± 0.08	0.86 ± 0.06	0.86 ± 0.00	ns	ns	ns
Isovaleric acid	1.41 ± 0.01	1.35 ± 0.18	1.16 ± 0.15	2.59 ± 0.03	2.84 ± 0.20	2.81 ± 0.01	1.25 ± 0.10	1.29 ± 0.05	1.30 ± 0.03	ns	ns	ns
∑ Volatile fatty acids C4-C5	5.79 ± 0.59	5.05 ± 0.35	4.79 ± 0.19	7.94 ± 0.35	8.45 ± 0.64	7.84 ± 0.19	**3.86 ± 0.01 a**	**3.68 ± 0.10 a**	4.64 ± 0.11 b	ns	ns	ns
Hexanoic acid	1.96 ± 0.27	2.27 ± 0.12	2.12 ± 0.36	2.94 ± 0.04	2.78 ± 0.15	2.75 ± 0.23	2.05 ± 0.22	2.22 ± 0.04	1.95 ± 0.09	ns	ns	ns
Octanoic acid	1.84 ± 0.24	2.23 ± 0.22	1.88 ± 0.24	2.75 ± 0.31	2.36 ± 0.01	2.71 ± 0.32	1.97 ± 0.06	2.33 ± 0.06	2.05 ± 0.28	ns	ns	ns
Decanoic acid	0.39 ± 0.15	0.43 ± 0.06	0.34 ± 0.66	1.22 ± 0.08	0.99 ± 0.03	1.12 ± 0.10	0.62 ± 0.06	0.69 ± 0.05	0.59 ± 0.02	ns	ns	ns
∑ Volatile fatty acids C6-C10	4.19 ± 0.66	4.93 ± 0.41	4.33 ± 0.65	6.91 ± 0.43	6.14 ± 0.19	6.58 ± 0.65	4.64 ± 0.22	5.24 ± 0.08	4.58 ± 0.39	ns	ns	ns
**Free terpenes (µg L^−1^)**												
trans-linalool oxide (furan) †	7.0 ± 0.8	5.4 ± 0.9	6.2 ± 0.1	5.2 ± 0.9	3.9 ± 0.3	4.1 ± 0.5	7.9 ± 0.2	7.8 ± 0.1	8.1 ± 0.7	ns	ns	ns
cis-linalool oxide (furan) †	0.7 ± 0.0	0.7 ± 0.2	0.8 ± 0.1	0.9 ± 0.0	0.6 ± 0.1	0.7 ± 0.1	1.1 ± 0.0	0.7 ± 0.1	0.8 ± 0.2	ns	ns	ns
trans-linalool oxide (pyran) †	4.5 ± 0.1	4.1 ± 0.1	4.0 ± 0.0	**4.7 ± 0.2 b**	**4.2 ± 0.1 ab**	**3.4 ± 0.3 a**	4.9 ± 2.5	4.9 ± 0.1	4.6 ± 0.7	ns	ns	ns
cis-linalool oxide (pyran) †	1.2 ± 0.2	0.9 ± 0.0	0.9 ± 0.1	1.1 ± 0.2	1.1 ± 0.2	0.9 ± 0.2	1.1 ± 0.1	0.9 ± 0.1	1.1 ± 0.5	ns	ns	ns
Linalool (L)	1.5 ± 0.0	1.4 ± 0.0	1.3 ± 0.2	3.6 ± 0.6	3.7 ± 0.7	3.4 ± 0.2	6.3 ± 0.2	6.2 ± 0.6	5.3 ± 0.5	ns	***	ns
Hotrienol †	0.5 ± 0.0	0.6 ± 0.0	0.6 ± 0.0	0.9 ± 0.2	1.1 ± 0.3	1.8 ± 0.6	1.3 ± 0.2	1.0 ± 0.1	1.3 ± 0.2	ns	*	ns
A-terpineol (αT)	2.2 ± 0.3	2.4 ± 0.1	2.3 ± 0.0	3.2 ± 0.0	2.8 ± 0.1	3.3 ± 0.3	4.5 ± 0.1	3.9 ± 0.7	3.1 ± 1.3	ns	**	ns
Citronellol (C)	<LOD	<LOD	<LOD	**8.1 ± 1.4 b**	**5.2 ± 0.4 a**	**4.2 ± 0.6 a**	**7.5 ± 0.6 b**	**6.3 ± 0.1 ab**	**5.7 ± 0.3 a**	*	ns	ns
Nerol (N)	<LOD	<LOD	<LOD	0.2 ± 0.2	0.2 ± 0.1	0.7 ± 0.3	2.2 ± 0.1	2.6 ± 0.4	2.7 ± 0.6	ns	***	ns
Geraniol (G)	<LOD	<LOD	<LOD	5.6 ± 1.1	5.5 ± 0.2	6.1 ± 0.5	6.8 ± 0.4	7.3 ± 0.9	6.3 ± 0.1	ns	ns	ns
Σ Free terpenes (L + αT + C + N + G)	3.6 ± 0.3	3.8 ± 0.4	3.6 ± 0.4	20.7 ± 1.7	17.2 ± 0.8	17.8 ± 1.1	27.3 ± 1.2	26.3 ± 1.1	23.1 ± 0.9	ns	***	ns
Hodiol I †	<LOD	<LOD	<LOD	5.4 ± 0.5	5.5 ± 0.5	5.9 ± 0.6	**5.7 ± 0.0 c**	**4.3 ± 0.0 b**	**3.7 ± 0.2 a**	ns	*	*
2,7-dimethyloctane-4,5-diol †	81.2 ± 6.4	111.0 ± 17.8	91.5 ± 12.5	160.9 ± 2.7	169.3 ± 15.1	152.7 ± 27.6	86.3 ± 17.3	76.0 ± 4.3	64.5 ± 5.3	ns	***	ns

Different letters indicate significant differences between treatments at *p* < 0.05 for a given compound or sum of compounds on a given year; for a rapid identification, these values appear in bold. The significance of the year, treatment and their interaction is expressed as ns = not significant; * *p* < 0.05; ** *p* < 0.01; *** *p* < 0.001. LOD means limit of detection. † indicates that the compound is expressed in µg L^−1^ of internal standard.

**Table 7:** Irrigation effects on the odor activity values (mean ± standard error) of Godello wines. Odor thresholds and descriptors for each compound are displayed. The significances of the year, treatment as well as their interaction are also shown. R = rain-fed, DI = drip irrigation, SDI = subsurface drip irrigation, T = Treatment, Y = Year.

	Odor Threshold (µg L^−1^)	Odor Descriptor	2012			2013			2014			T	Y	T × Y
			R	DI	SDI	R	DI	SDI	R	DI	SDI			
Ethyl acetate	7500	Pineapple	8 ± 1	7 ± 1	8 ± 0	4 ± 0	3 ± 0	4 ± 0	5 ± 1	5 ± 0	5 ± 0	ns	*	ns
Acetaldehyde	500	Fruity	116 ± 25	77 ± 2	56 ± 4	62 ± 6	71 ± 7	61 ± 4	62 ± 12	55 ± 1	49 ± 1	*	**	ns
**Higher alcohols**														
1-propanol	750	Alcohol	16 ± 0	17 ± 1	16 ± 3	**29 ± 1 ab**	**32 ± 0 b**	**26 ± 0 a**	31 ± 6	32 ± 5	22 ± 3	ns	**	ns
2-methyl-1-propanol	40000	Alcohol	2 ± 0	1 ± 0	2 ± 0	1 ± 0	1 ± 0	1 ± 0	1 ± 0	1 ± 0	1 ± 0	ns	***	ns
3-methyl-1-butanol	30000	Alcohol	8 ± 1	9 ± 1	9 ± 0	10 ± 0	10 ± 0	10 ± 0	7 ± 0	7 ± 0	8 ± 0	ns	ns	ns
**Other alcohols**														
*cis*-3-hexen-1-ol	400	Grass	1 ± 0	1 ± 0	1 ± 0	1 ± 0	1 ± 0	1 ± 0	1 ± 0	1 ± 0	<1	ns	**	ns
Benzyl alcohol	620	Blackberry	**6 ± 0 b**	**4 ± 0 a**	**4 ± 0 a**	4 ± 0	4 ± 0	3 ± 0	**3 ± 0 a**	**4 ± 0 ab**	**5 ± 0 b**	ns	**	**
2-phenylethanol	14000	Rose	4 ± 0	4 ± 0	3 ± 0	3 ± 0	4 ± 0	4 ± 0	**2 ± 0 a**	**3 ± 0 b**	**2 ± 0 a**	ns	**	ns
**Acetates of higher alcohols**														
Isoamyl acetate	30	Banana	24 ± 1	22 ± 1	17 ± 3	42 ± 13	45 ± 12	26 ± 1	25 ± 3	28 ± 1	29 ± 2	ns	ns	ns
**Esters**														
Ethyl butyrate	20	Fruity	2 ± 0	2 ± 0	2 ± 1	<1	<1	<1	3 ± 0	3 ± 0	3 ± 0	ns	ns	ns
Ethyl hexanoate	14	Fruity	19 ± 1	21 ± 1	21 ± 6	31 ± 2	26 ± 1	28 ± 2	18 ± 2	15 ± 0	15 ± 0	ns	ns	ns
Ethyl octanoate	5	Fruity	67 ± 1	82 ± 2	76 ± 16	141 ± 13	108 ± 14	120 ± 22	59 ± 1	67 ± 1	65 ± 9	ns	ns	ns
Ethyl decanoate	200	Grape	<1	<1	<1	2 ± 0	1 ± 0	1 ± 0	1 ± 0	1 ± 0	1 ± 0	ns	ns	ns
**Volatile fatty acids**														
Isobutyric acid	2300	Cheese	1 ± 0	1 ± 0	1 ± 0	2 ± 0	2 ± 0	2 ± 0	**<1 a**	**<1 a**	**1 ± 0 b**	ns	ns	ns
Butyric acid	173	Cheese	6 ± 1	5 ± 0	5 ± 0	9 ± 0	8 ± 0	8 ± 0	5 ± 0	5 ± 0	5 ± 0	ns	ns	ns
Isovaleric acid	33	Cheese	43 ± 0	41 ± 5	35 ± 5	78 ± 1	86 ± 6	85 ± 0	38 ± 3	39 ± 2	39 ± 1	ns	ns	ns
Hexanoic acid	3000	Cheese	1 ± 0	1 ± 0	1 ± 0	1 ± 0	1 ± 0	1 ± 0	1 ± 0	1 ± 0	1 ± 0	ns	ns	ns
Octanoic acid	500	Rancid	4 ± 0	4 ± 0	4 ± 0	6 ± 1	5 ± 0	5 ± 1	4 ± 0	5 ± 0	4 ± 1	ns	ns	ns
Decanoic acid	1000	Rancid	<1	<1	<1	1 ± 0	1 ± 0	1 ± 0	1 ± 0	1 ± 0	1 ± 0	ns	ns	ns

Different letters indicate significant differences between treatments at *p* < 0.05 for a given compound on a given year; for a rapid identification, these values appear in bold. The significance of the year, treatment and their interaction is expressed as ns = not significant; * *p* < 0.05; ** *p* < 0.01; *** *p* < 0.001. Odor thresholds have been taken from Guth [22] and Ferreira et al. [23].

## Supplementary Materials

The following are available online at https://www.mdpi.com/2304-8158/8/4/135/s1, Figure S1: Visual and palate descriptors for Godello wines (averaged from 2012 to 2014) as a function of rain-fed and irrigation conditions, Table S1: Irrigation effects on the amino acid concentrations (mean ± standard error, mg L^−1^) of Godello wines.
