# Supplementary material for: Effects of Two Different Irrigation Systems on the Amino Acid Concentrations, Volatile Composition and Sensory Profiles of Godello Musts and Wines
Source: Foods. 2019 Apr 22;8(4):135. doi: 10.3390/foods8040135 (PMC6517885; doi:10.3390/foods8040135)

Supplementary Table S1. Irrigation effects on the amino acid concentrations (mean ± standard error, mg L^{-1}) of Godello wines. The significances of the factors year and treatment, as well as their interaction are also shown. The concentration of proline is not considered for calculating the sum of amino acids. R = rain-fed, DI = drip irrigation, SDI = subsurface drip irrigation.

Compound	2012			2013			2014			Treatment	Year	Treatment x Year
	R	DI	SDI	R	DI	SDI	R	DI	SDI			
**Aspartic acid**	12.2 ± 2.9	14.4 ± 3.2	12.1 ± 1.9	13.0 ± 0.6	12.4 ± 0.5	10.8 ± 0.5	24.6 ± 5.2	22.4 ± 2.4	14.2 ± 0.4	ns	**	ns
**Glutamic acid**	1.7 ± 1.7	2.1 ± 0.4	2.5 ± 0.1	10.8 ± 1.1	5.1 ± 4.6	0.7 ± 0.5	11.6 ± 2.3	12.9 ± 0.4	8.3 ± 0.7	ns	***	ns
**Asparagine**	2.8 ± 1.1	4.5 ± 1.5	3.1 ± 0.1	7.7 ± 0.6	7.7 ± 0.0	6.0 ± 0.2	12.2 ± 4.6	12.3 ± 0.6	7.6 ± 0.7	ns	***	ns
**Serine**	3.1 ± 1.0	3.0 ± 0.5	2.4 ± 0.2	**4.1 ± 0.2 b**	**4.1 ± 0.0 b**	**3.2 ± 0.0 a**	**5.9 ± 0.4 b**	**5.2 ± 0.1 b**	**3.8 ± 0.1 a**	**	***	ns
**Glutamine**	0.2 ± 0.1	0.4 ± 0.1	0.2 ± 0.0	0.6 ± 0.0	0.6 ± 0.0	0.4 ± 0.1	0.6 ± 0.1	0.6 ± 0.0	0.3 ± 0.0	*	**	ns
**Histidine**	2.8 ± 0.7	4.2 ± 1.0	3.3 ± 0.8	5.9 ± 0.6	4.2 ± 1.4	4.0 ± 0.6	5.9 ± 1.0	6.1 ± 0.2	4.2 ± 0.0	ns	*	ns
**Glycine**	3.6 ± 0.5	4.1 ± 0.5	3.1 ± 0.4	**4.2 ± 0.4 b**	**3.5 ± 0.5 b**	**2.3 ± 0.1 a**	**3.8 ± 0.2 b**	**3.1 ± 0.2 b**	**2.3 ± 0.0 a**	**	ns	ns
**Threonine**	2.1 ± 0.6	2.1 ± 0.5	1.9 ± 0.2	2.2 ± 0.2	2.4 ± 0.1	2.1 ± 0.0	**3.5 ± 0.1 b**	**2.9 ± 0.2 ab**	**2.3 ± 0.1 a**	ns	**	ns
**Arginine**	4.3 ± 2.3	5.3 ± 1.7	4.6 ± 0.5	7.1 ± 1.0	7.5 ± 0.3	6.1 ± 0.2	8.7 ± 1.6	8.0 0.6±	5.9 ± 0.1	ns	**	ns
**Alanine**	7.9 ± 3.2	6.8 ± 1.9	5.0 ± 0.3	7.7 ± 0.5	7.6 ± 0.1	6.3 ± 0.1	9.4 ± 2.3	8.5 ± 0.5	6.4 ± 0.6	ns	ns	ns
**γ-Aminobutyric acid (GABA)**	5.4 ± 3.8	4.0 ± 1.2	2.8 ± 0.0	8.4 ± 0.8	7.8 ± 0.2	6.1 ± 0.4	10.8 ± 7.5	8.0 ± 1.7	3.1 ± 0.5	ns	ns	ns
**Proline**	379.8 ± 134.5	824.1 ± 222.4	489.4 ± 287.2	895.4 ± 297.9	854.9 ± 377.8	454.7 ± 251.6	371.4 ± 344.6	761.1 ± 111.5	237.9 ± 203.6	ns	ns	ns
**Tyrosine**	2.3 ± 1.2	2.7 ± 0.9	2.2 ± 0.4	**3.4 ± 0.1 b**	**3.6 ± 0.1 b**	**2.9 ± 0.1 a**	0.9 ± 0.2	0.9 ± 0.2	0.9 ± 0.0	ns	*	ns
**Ammonium ion**	3.7 ± 1.0	5.1 ± 0.6	4.4 ± 0.7	4.8 ± 0.7	4.2 ± 0.2	3.9 ± 0.5	6.0 ± 1.4	5.9 ± 0.4	4.6 ± 0.4	ns	ns	ns
**Valine**	3.8 ± 2.4	4.2 ± 1.6	3.0 ± 0.2	6.5 ± 0.1	7.1 ± 0.3	5.6 ± 0.4	**9.3 ± 0.2 b**	**6.5 ± 0.7 ab**	**4.2 ± 0.9 a**	ns	**	ns
**Methionine**	1.4 ± 0.6	1.6 ± 0.5	1.3 ± 0.1	1.6 ± 0.0	1.5 ± 0.1	1.2 ± 0.1	3.1 ± 0.4	2.7 ± 0.2	1.8 ± 0.0	ns	**	ns
**Cysteine**	0.0 ± 0.0	0.0 ± 0.0	0.0 ± 0.0	0.4 ± 0.2	0.0 ± 0.0	0.0 ± 0.0	0.0 ± 0.0	0.0 ± 0.0	0.0 ± 0.0	ns	ns	ns
**Isoleucine**	1.8 ± 0.8	1.9 ± 0.5	1.4 ± 0.0	2.5 ± 0.0	2.7 ± 0.0	2.1 ± 0.3	**3.9 ± 0.2 b**	**2.8 ± 0.3 ab**	**2.0 ± 0.3 a**	*	**	ns
**Tryptophan**	1.1 ± 0.2	1.1 ± 0.1	1.2 ± 0.0	0.6 ± 0.2	0.7 ± 0.1	0.5 ± 0.0	0.9 ± 0.1	1.0 ± 0.1	0.8 ± 0.0	ns	ns	ns
**Leucine**	5.2 ± 3.1	5.9 ± 2.1	4.2 ± 0.2	8.3 ± 0.5	8.5 ± 0.1	7.0 ± 0.0	**12.1 ± 1.6b**	**9.8 ± 0.8ab**	**6.3 ± 0.4a**	ns	**	ns
**Phenylalanine**	3.6 ± 2.0	4.0 ± 1.3	3.0 ± 0.1	5.0 ± 0.3	5.3 ± 0.3	4.7 ± 0.0	7.7 ± 1.3	6.7 ± 0.5	4.3 ± 0.3	ns	**	ns
**Ornithine**	0.9 ± 0.4	0.6 ± 0.1	0.7 ± 0.3	0.5 ± 0.0	0.6 ± 0.0	0.7 ± 0.1	0.9 ± 0.4	1.2 ± 0.3	1.8 ± 0.8	ns	ns	ns
**Lysine**	6.0 ± 3.2	8.3 ± 2.9	7.1 ± 1.1	11.2 ± 1.5	11.7 ± 0.3	9.1 ± 0.0	15.1 ± 2.8	12.8 ± 1.0	8.1 ± 0.0	ns	**	ns
**Sum of amino acids**	72.5 ± 30.8	81.2 ± 22.7	65.1 ± 5.9	**111.8 ± 5.1 b**	**104.5 ± 5.4 b**	**81.8 ± 0.3 a**	150.8 ± 31.8	134.3 ± 10.4	88.5 ± 0.4	*	**	ns

Different letters indicate significant differences between treatments at $p < 0.05$ for a given amino acid in a given year; for a rapid identification, these values appear in bold. The significance of the year, treatment and their interaction is expressed as ns = non-significant; * $p < 0.05$; ** $p < 0.01$; *** $p < 0.001$.

Supplementary Figure S1. Visual and palate descriptors for Godello wines (averaged from 2012 to 2014) as a function of rain-fed and irrigation conditions. R = Rain-fed, DI = Drip irrigation, SDI = Subsurface drip irrigation.

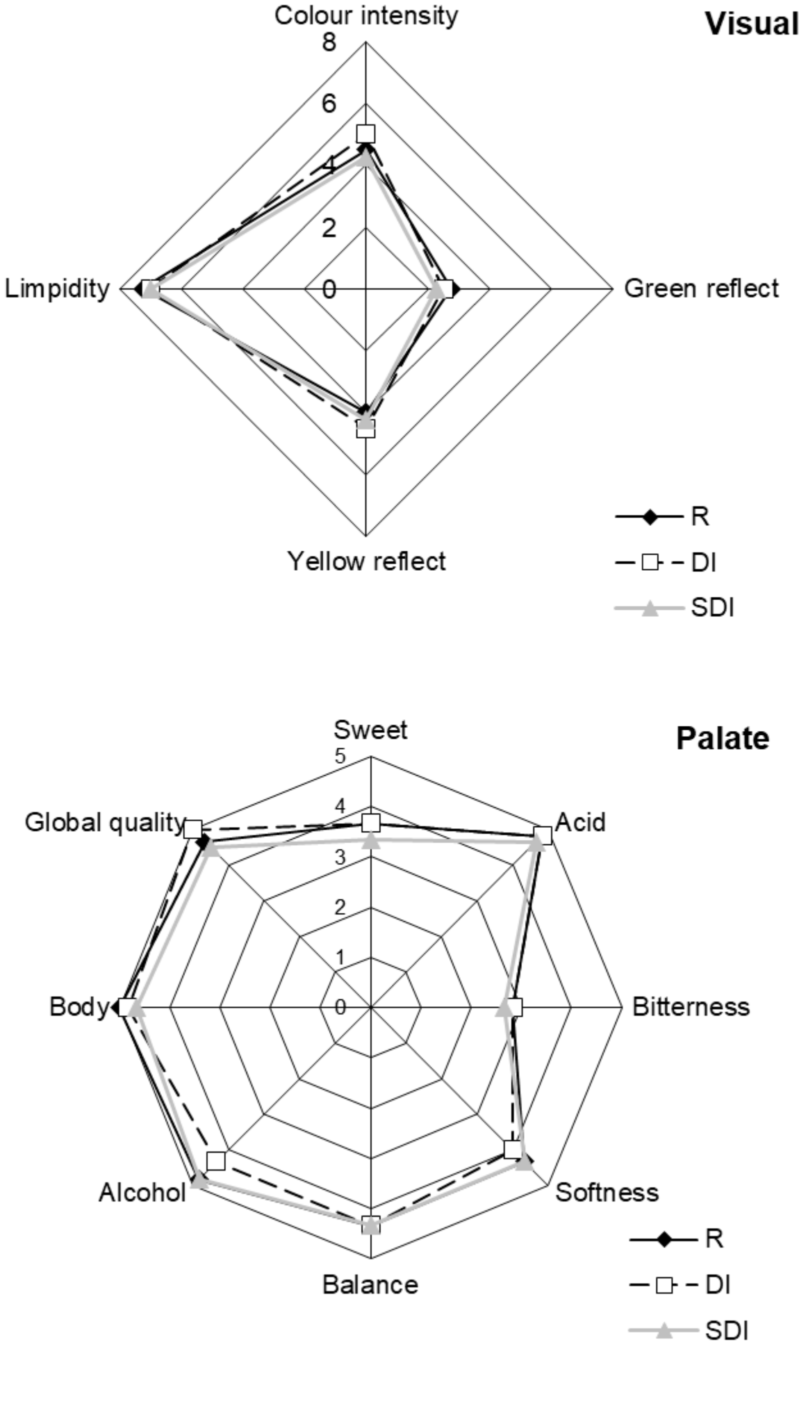

Supplement: Supplementary file 1 [file foods-08-00135-s001.pdf]
